# Influence of Environmental Conditions on the Fusion of Cationic Liposomes with Living Mammalian Cells

**DOI:** 10.3390/nano9071025

**Published:** 2019-07-17

**Authors:** Rejhana Kolašinac, Sebastian Jaksch, Georg Dreissen, Andrea Braeutigam, Rudolf Merkel, Agnes Csiszár

**Affiliations:** 1 Forschungszentrum Jülich GmbH, Institute of Complex Systems: ICS-7 Biomechanics, 52428 Jülich, Germany; 2 Forschungszentrum Jülich GmbH, Jülich Centre for Neutron Science (JCNS) at Heinz Maier-Leibnitz Zentrum (MLZ), 85748 Garching, Germany; 3 Forschungszentrum Jülich GmbH, Institute of Complex Systems: ICS-2 Theoretical Soft Matter and Biophysics, 52428 Jülich, Germany

**Keywords:** cationic liposomes, membrane fusion, lipid phases, fusion conditions

## Abstract

Lipid-based nanoparticles, also called vesicles or liposomes, can be used as carriers for drugs or many types of biological macromolecules, including DNA and proteins. Efficiency and speed of cargo delivery are especially high for carrier vesicles that fuse with the cellular plasma membrane. This occurs for lipid mixture containing equal amounts of the cationic lipid DOTAP and a neutral lipid with an additional few percents of an aromatic substance. The fusion ability of such particles depends on lipid composition with phosphoethanolamine (PE) lipids favoring fusion and phosphatidyl-choline (PC) lipids endocytosis. Here, we examined the effects of temperature, ionic strength, osmolality, and pH on fusion efficiency of cationic liposomes with Chinese hamster ovary (CHO) cells. The phase state of liposomes was analyzed by small angle neutron scattering (SANS). Our results showed that PC containing lipid membranes were organized in the lamellar phase. Here, fusion efficiency depended on buffer conditions and remained vanishingly small at physiological conditions. In contrast, SANS indicated the coexistence of very small (~50 nm) objects with larger, most likely lamellar structures for PE containing lipid particles. The fusion of such particles to cell membranes occurred with very high efficiency at all buffer conditions. We hypothesize that the altered phase state resulted in a highly reduced energetic barrier against fusion.

## 1. Introduction

Artificial vesicles or liposomes are frequently investigated due to many applications in pharmacology and medicine. They have been used as carrier particles for various compounds, e.g., DNA, RNA, proteins, and anti-cancer therapeutics [1,2]. Cargo delivery can be substantially improved using vesicle carriers that are able to fuse with the first cellular barrier, the plasma membrane. To this end, viral membranes, which naturally contain fusogenic, membrane-associated peptides [1,3,4] and peptide-free cationic liposomes have been used with similar high-fusion efficiency [2]. In viral carriers, proteins embedded into the lipid bilayer or associated with it are directly involved in fusion induction, as well as in processes that precede or follow membrane coalescence [5,6,7,8]. Synthetic liposomes can also benefit from fusion peptides and proteins [9,10], but their presence is not mandatory for efficient membrane fusion induction. Cholesterol [11], as well as DNA in lipoplexes [12], have also been reported as membrane fusion initiators. Moreover, pure lipid bilayers assembled from phosphoethanolamines, for example, or its derivatives, are also known in the literature as fusogenic [13,14]. Such phospholipids, in general, have a small head group and long unsaturated chains with an inverse conical molecular shape, preferably forming three-dimensional phases like inverse hexagonal (H_II_) or cubic instead of a lamellar phase. During the phase transition from one into the other phase, a fusion-intermediate structure can form [14,15,16,17,18,19]. In some cases, two-dimensional lipid mixing entropy is the driving force regulating the spontaneous formation of multicomponent membranes [20].

Although such liposomes prevailed as fusogenic, they reach significant fusion efficiencies with living cells only in combination with a cationic lipid, e.g., DOTAP, and an aromatic molecule, such as fluorescence dyes [21]. Such so-called fusogenic liposomes (FLs) are able to fuse with mammalian cells with high efficiency of about 80–100% within 1–5 min without any toxic effect. As shown previously, the cargo molecules, e.g., proteins [22], nucleic acids [23], or different kind of lipids [24], can efficiently be delivered to many different cell types due to successful membrane mixing of the liposomal and the plasma membranes and subsequent content release. Here, membrane fusion success correlated with the delivery efficiency of the cargo. Therefore, we systematically varied the composition of empty FLs and identified the components essential for fusion with biological membranes [25]. Thereby, we found that a cationic lipid with an inverted conical shape and an aromatic molecule are mandatory for fusion induction. Although the addition of neutral lipids, e.g., DPPC or DOPE, is not essential, it can be used to tune fusion efficiency. The simple exchange of DOPE with DOPC can turn the fusion ability of liposomes from 90% into 10% without nominative changes either in particle size or zeta potential characteristic for the surface charge of the liposomes.

The central event during membrane fusion is the merging of two membranes, which is defined by the lipid matrix itself. Beside embedded proteins and lipid composition, environmental conditions determine fusion ability. For example, Zimmerberg and coworkers examined membrane fusion in a living cell system and found that it is driven by an osmotic gradient [26], while Akimov et al. showed for a protein-free model system that changes of the environmental pH in the physiologically relevant range between 4.0 and 7.5, notably affected the membrane fusion rate [27].

Because membrane fusion, in general, is in many instances dependent on environmental conditions we systematically studied the role of temperature, osmolality, pH and ionic concentration of the buffer on the fusion efficiency of cationic liposomes with living cells in vitro. Chinese hamster ovary (CHO) cells were used as mammalian cells, and fusion efficiency of cationic liposomes containing phosphoethanolamine (PE) or phosphatidyl-choline (PC) as neutral compounds and an aromatic molecule were analyzed. Additionally, structural investigation of liposomes was carried out using small angle neutron scattering (SANS) [28] to study the influence of thermotropic lipid phases for efficient membrane fusion.

## 2. Materials and Methods

### 2.1. Chemicals

We used the cationic lipid 1,2-dioleoyl-3-trimethylammonium-propane (chloride salt) (DOTAP), and the neutral lipids 1,2-dipalmitoyl-sn-glycero-3-phosphoethanolamine (C16(0)PE), 1,2-dipalmitoleoyl-sn-glycero-3-phosphoethanolamine (C16(1)PE), 1,2-dipalmitoyl-sn-glycero-3-phosphocholine (C16(0)PC), 1,2-dipalmitoleoyl-sn-glycero-3-phosphocholine (C16(1)PC), 1,2-distearoyl-sn-glycero-3-phosphoethanolamine (C18(0)PE), 1,2-dioleoyl-sn-glycero-3-phosphoethanolamine (C18(1)PE), 1,2-distearoyl-sn-glycero-3-phosphocholine (C18(0)PC), and 1,2-dioleoyl-sn-glycero-3-phosphocholine (C18(1)PC). As fluorescently labeled lipids 1,2-dioleoyl-sn-glycero-3-phosphoethanolamine-N-(dipyrrometheneborondifluoride)butanoyl (TFPE-head) and 1-palmitoyl-2-(dipyrrometheneboron difluoride)-undecanoyl-sn-glycero-3-phosphoethanolamine (TFPE-chain) were applied. All mentioned lipids were purchased from Avanti Polar Lipids, Inc. (Alabaster, AL, USA) and used without further purification. The fluorescently labelled lipid N-(4,4-difluoro-5,7-dimethyl-4-bora-3a,4a-diaza-s-indacene-3-propionyl)-1,2-dihexadecanoyl-sn-glycero-3-phosphoethanolamine (triethylammonium salt) (BODIPY FL-DHPE) and the lipid analogue 1,1′-dioctadecyl-3,3,3′,3′-tetramethylindotricarbocyanine iodide also called DiIC_18_(7) (DiR) was ordered from Thermo Fisher Scientific (Eugene, OR, USA).

### 2.2. Preparation of Liposomes

#### 2.2.1. Liposomes for Treatment of CHO Cells and Microscopy

Liposomes were prepared according to the method described by Kolasinac et al. with few modifications [25]. In brief, lipid components, like neutral and cationic lipids, and the fluorescent compound were mixed in chloroform (EMSURE grade, VWR, Darmstadt, Germany) at a ratio of 1/1/0.1 mol/mol. Chloroform was evaporated under vacuum for 0.5 h. Afterward, lipids were dispersed in 20 mM N-2-hydroxyethylpiperazine-N-2 ethane sulfonic acid (HEPES) buffer (VWR, Darmstadt, Germany) at a total lipid concentration of 2 mg/mL and pH 7.4 (osmolality 30 mOsm/kg) or in distilled and deionized water at the same concentration. Buffer osmolality was determined using a freezing point osmometer (Osmomat 030 from Gonotec, Berlin, Germany). The solution was vortexed for 1–2 min to produce multilamellar liposomes. After homogenization in an ultrasonic bath (Sonocool, Bandelin electronic GmbH, Berlin, Germany) for 20 min at 5 °C, mainly unilamellar vesicles were formed. Before usage, liposomes were kept at 4 °C for no longer than two days. 

#### 2.2.2. Liposomes for Small Angle Neutron Scattering (SANS)

For SANS, preparation of liposomes was slightly altered. For these experiments, BODIPY-FL-DHPE was used as a dye. The total lipid concentration was set to 10 mg/mL. After evaporation of chloroform, the lipid film was resuspended in 20 mM HEPES dissolved in D_2_O (99 atom % D, Sigma-Aldrich, Taufkirchen, Germany) and vortexed vigorously without additional sonication. Samples were stored at −20 °C until usage. One hour before measurements, samples were thawed and vortexed vigorously before being transferred into quartz cuvettes (110-QS, quartz glass, Suprasil, 1 mm path length, Hellma, Müllheim, Germany) for SANS measurements.

### 2.3. Cell Culture

Experiments were performed on Chinese Hamster Ovary K1 cells (CHOs) purchased from American Type Culture Collection (ATTC, Manassas, VA, USA). They were maintained in DMEM-F12 (Sigma-Aldrich, Taufkirchen, Germany) supplemented with 10% fetal bovine serum (FBS) (Thermo Fisher Scientific, Waltham, MA, USA), 10,000 units penicillin and 10 mg/mL streptomycin (both Sigma-Aldrich). During culture, cells were kept at 37 °C and 5% CO_2_ in a saturated humid atmosphere. Cell density never exceeded 80% confluence. Before microscopy, glass surfaces (∅ = 3.5 cm Petri dish) were coated with human fibronectin (10 µg/mL, BD Biosciences, San Jose, CA, USA) for 30 min at 37 °C and 50,000 cells were seeded on them and cultivated for 24 h. Cell nuclei were stained for 15 min at 37 °C with DRAQ5 (red fluorescence, Thermo Fischer Scientific, Waltham, MA, USA) or Hoechst 33342 (blue fluorescence, Thermo Fischer Scientific, Waltham, MA, USA) according to the manufacturer’s protocols. Furthermore, 10 µL of the liposome stock solutions were diluted 1/50 with phosphate buffer saline (PBS, 290 mOsm/kg, 137 mM NaCl; 2.7 mM KCl; 1.47 mM KH_2_PO_4_; 8.1 mM Na_2_HPO_4_) at pH 5–9 (adjusted by addition of 1 M NaOH or HCl), phosphate buffer (PB, 30 mOsm/kg, 2.7 mM KCl; 1.47 mM KH_2_PO_4_; 8.1 mM Na_2_HPO_4_), or glucose solution of defined osmolality (30 mOsm/kg and 290 mOsm/kg) and cells on glass substrates (~100,000 of cells) were incubated in these solutions for 5 min in the incubator (37 °C and 5% CO_2_, saturated humid atmosphere). After the treatment, the suspension of liposomes was exchanged with fresh, pre-warmed cell culture medium. In some control experiments, cells were first washed three times using a sodium heparin solution at 2 mg/mL concentration in PBS buffer before medium exchange to eliminate remaining liposomes from the cell surfaces. For temperature-dependent experiments, 10 µL of the liposomal stock solutions, stored at 4 °C no longer than 24 h, were diluted 1/50 with phosphate buffer saline (PBS, 290 mOsm/kg) at room temperature. Before, liposomes and cells were left for 5 min at room temperature. Immediately afterward, the cells were incubated with liposomal solutions at 4 °C, 20 °C, 30 °C or 37 °C for 5 min. Thereafter, they were washed by fresh, pre-warmed cell culture medium, and samples were immediately analyzed by laser scanning microscopy.

### 2.4. Characterization of Size and Zeta Potential Distribution of Liposomes

Both particle size and ζ-potential distributions were measured using a zetasizer (Nano ZS from Malvern Instruments, Malvern, UK) equipped with a HeNe laser (633 nm). Scattered laser light was collected at a constant angle of 173°. Prior to measurements liposome stock solutions were diluted using the appropriate buffer. The temperature was set using the instrument thermostat. Data were collected from three independently prepared samples and analyzed using the instrument software (DTS from Malvern Instruments). Reported data are mean peak position and its standard deviation (mean (s.d.))

### 2.5. Microscopy

Samples were imaged using a confocal laser scanning microscope (LSM 710 from Carl Zeiss MicroImaging GmbH, Jena, Germany) equipped with a near UV laser (405 nm), an argon ion laser (488 nm), and a helium-neon laser (633 nm). Both TopFluor lipid derivatives were excited at 488 nm, and their fluorescence emissions were detected using a bandpass filter BP 495–550 nm (green channel). The lipid analog DiR and the nuclear stain DRAQ5 were excited using the 633 nm laser line, and the emitted signal was collected through the long pass filter LP 650 nm. Hoechst 33342 was excited by the 405 nm laser line, and its emission was detected using a BP 505/90. For imaging, a Plan-Apochromat 40×/1.40 Ph3 (Carl Zeiss, Oberkochen, Germany) objective was used. To maintain appropriate culture conditions, the confocal microscope was equipped with an incubator (Incubator XL 2, Carl Zeiss, Oberkochen, Germany). Temperature and CO_2_ were kept constant at 37 °C and 5%, respectively. The experiments were repeated at least three times. For each experiment minimum five images were taken, with at least one hundred cells per image. Overall, fusion efficiency was determined from no less than 1500 cells at each condition.

### 2.6. Analysis of Images 

An algorithm was developed to quantify fusion efficiency from fluorescence micrographs. It is based on staining intensity. The code was implemented in Matlab (R2017, Mathworks, Natick, MA, USA). In the first step, individual cells were segmented. To this end, the nuclei channel was used. This image was first smoothed (Gaussian filter, standard deviation 3 pixels, pixel size 173 nm throughout) and morphological opening (disk-shaped structuring element of radius 9 pixels) was performed. The bright nuclei were segmented using the mean grey value of the image as an intensity threshold. Subsequently, morphological opening and closing (both with a disk of radius 5 pixels) were performed on the mask. Next, the watershed transformation was used to separate overlapping nuclei and to segment cells. Therefore, the distance transform of the negative mask was calculated and multiplied by −1. Then, local minima (depth less than 2) were eliminated from this resulting image, and finally, the watershed transform was applied to that image. Next, to the separated nuclei labels, the dividing lines between the “watershed” areas of neighboring nuclei, computed by the watershed transform, were used as the shape of the corresponding cell.

Afterward, the fluorescent lipid signal was analyzed. Since the many small, bright spots in the images indicated lumps, endocytic, or non-fused liposomes, they were removed. Therefore, the image was first smoothed (Gaussian filter, the standard deviation of 3 pixels). Then, local bright spots were detected using the algorithm described in the work of Hersch and colleagues [29]. In brief, the local z-score of each pixel was calculated within a 91 × 91 environment and segmented using a threshold of 2 for the z-score. Only regions with the area below 100,000 pixels × pixels were accepted. Each such region was then enlarged by morphological dilation (the disk of radius 3 pixels). On the smoothed fusion signal image, all spots identified by the z-score segmentation were replaced by pixel values calculated by inward interpolation from the grey scale values at the rim of the spot (MatLab function region fill). Spot detection and interpolation of grey values were performed twice. Using the processed intensity image from the second part of the program and the cell label image from the first part, the average grey value intensity for each cell was calculated individually. Using the same manually chosen threshold for all images, all cells were separated into fused (all cells with an average grey value above the threshold) and non-fused (all cells below the threshold) cells.

#### 2.6.1. Small Angle Neutron Scattering (SANS)

SANS experiments were carried out at small angle scattering set-up KWS-2, operated by JCNS at Forschungsneutronenquelle Heinz Maier-Leibnitz, FRM II, in Garching (Germany) [30]. A wavelength of λ = 7 Å (Δ λ/λ = 10%) and sample-detector distances (SDD) of 1.58, 7.58, and 19.48 m were used to cover a q-range of 0.002–0.221 Å^−1^. The detector was a ^3^He detector with a resolution of 8 mm. Exposure times were 5 min at SDD = 1.58 m, 10 min at SDD = 7.58, and 20 min at SDD = 19.48 m. Samples were placed in an aluminum holder with plastic cover, and the temperature was controlled by a Peltier element combined with a counter cooling by a water thermostat. The cuvettes were Hellma quartz glass cuvettes with a 1 mm sample thickness. Experiments were performed at 37 °C. The scattering of D_2_O and the empty cell were subtracted from the sample scattering taking the transmissions into account. The resulting intensities were azimuthally averaged. Good agreement was found wherever curves at different SDDs overlapped. All data corrections were performed with the software QtiKWS provided by JCNS. Fitting was done by SasView software version 4.2.0 (http://sasview.org).

#### 2.6.2. Model Functions Used for the Data Fitting

All fit functions contained a scale factor *I*_0_ and background *I_b_*, i.e., they were of the form

(1) $$I\left(q\right)\;=\;I_{0}f\left(q\right)+I_{b}$$

where *q* denotes the scattering vector. The scale factor contains the scattering volume and the scattering length density difference between solvent and structure.

The lamellar model provides the scattering intensity, *I*(*q*), for a lyotropic lamellar phase. A uniform scattering length density and random distribution in solution are assumed, which results in

(2) $$f\left(q\right)\;\propto \;\frac{1-\cos\left(q\delta \right)}{\delta q^{4}}$$

where *δ* denotes bilayer thickness [31].

The ellipsoid model is calculated from the form factor for randomly oriented ellipsoids of revolution with uniform scattering length density. This results in

(3) $$f\left(q\right)=\frac{{\left[\sin\left(qr\right)-qr\cos\left(qr\right)\right]}^{2}}{9{\left(qr\right)}^{6}}$$

with

(4) $$r=\sqrt{R_{b}{}^{2}\sin^{2}\alpha +R_{a}{}^{2}\cos^{2}\alpha }$$

where α denotes the angle between the rotational axis of the ellipsoid and the *q*-vector, *R_a_* is its radius along this axis, and *R_b_* the radius perpendicular to it. The orientation of the ellipsoid is numerically averaged over a sphere to give the final fit model [28,32].

As the final model, we used a general power law

(5) $$f\left(q\right)={\left\|q\right\|}^{-m}$$

### 2.7. Statistical Analysis

Statistical analyses of data were performed by one-way ANOVA using Origin 9.0 (OriginLab Co., Northampton, MA, USA). *p* < 0.01 was considered statistically significant. Data are expressed as means (s.d.).

## 3. Results

As shown recently, lipid composition strongly influences the fusion ability and fusion efficiency of liposomes [25]. Depending on their lipid composition, liposomes are either taken up by endocytosis or membrane fusion. Here, we compared liposomes with compositions characteristic for both uptake pathways. The analyzed liposomes always contained the cationic lipid DOTAP, neutral lipid, and a fluorescent dye at the molar ratio of 1/1/0.1 mol/mol. If the neutral lipid was a PC, endocytic uptake was expected. In the following, such liposomes will also be called “endocytic liposomes” or “Els”. However, liposomes containing a PE as neutral lipid were more able to fuse with cellular membranes [25] and are hence called “fusogenic liposomes” or “FLs”. Accordingly, PC and PE lipids with 16 and 18 carbon atoms (C16 or C18) with or without unsaturation in the fatty acid chains ((1) or (0), respectively) were tested as neutral compounds. Additionally, the Bodipy derivative TopFluor coupled at different positions to PE (TFPE) or the carbocyanine dye with long fatty acid chains DiR (for IUPAC names see Materials and Methods) were incorporated into the liposomes and tested for fusion induction.

**Figure 1:**
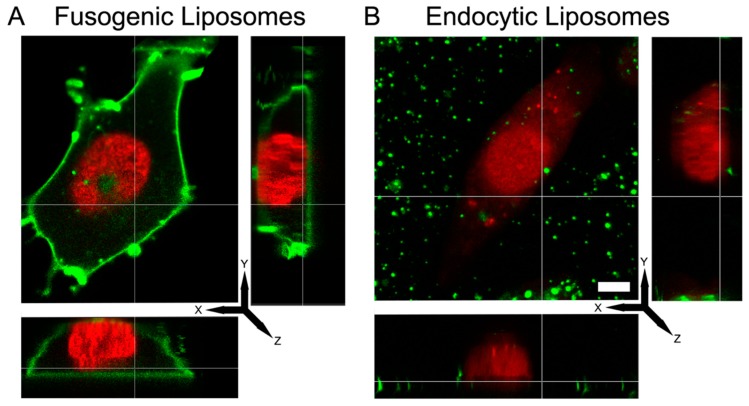
3D-fluorescence micrograph of a Chinese hamster ovary (CHO) cell treated with fusogenic liposomes (FLs) DOPE/DOTAP/TFPE-head (1/1/0.1 mol/mol) (**A**), and endocytic liposomes (ELs) DOPC/DOTAP/TFPE-head (1/1/0.1 mol/mol) (**B**). Green: TFPE-head signal, red: Nuclei staining with DRAQ5. Scale bar, 20 µm, applies to all.

To identify whether the different fluorescence signal patterns belonged to membrane fusion or endocytosis, 3D confocal imaging of CHO cells were carried out upon treatment with liposomes composed of DOPE/DOTAP/TopFluor-head (1/1/0.1 mol/mol) (FLs) or DOPC/DOTAP/TopFluor-head (1/1/0.1 mol/mol). As shown in Figure 1A, FLs homogeneously stained the whole cellular plasma membrane while ELs was localized mainly on the cell surface without distributing in the membrane (Figure 1B) or internalized in the cell cytoplasm. Signal distributions were not significantly influenced by a washing step using sodium heparin, a polyanionic solution (Figure S1).

### 3.1. Influence of Temperature

Liposomes with the composition described above were characterized from the physicochemical point of view depending on the temperature. Here, FLs showed a homogeneous population (see polydispersity index (PDI) in Table 1) with a hydrodynamic diameter of about 115 nm and zeta potential of + 50 mV without significant changes in the temperature range from 4 °C to 37 °C (see Table 1). Within statistical significance, endocytic liposomes (ELs) gave similar results as FLs (see Table 1).

**Figure 2:**
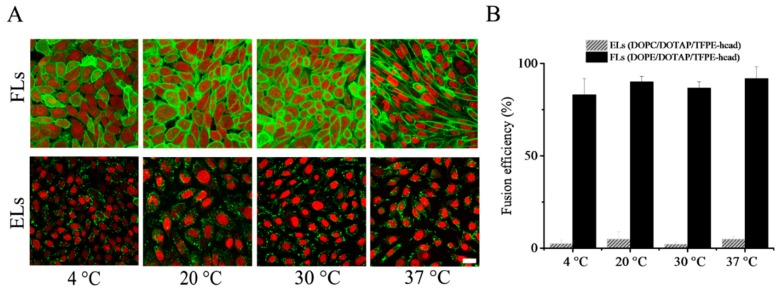
(**A**) Fluorescence micrographs of CHO cells treated with fusogenic liposomes (FLs) DOPE/DOTAP/TFPE-head (1/1/0.1 mol/mol), upper row, and endocytic liposomes (ELs) DOPC/DOTAP/TFPE-head (1/1/0.1 mol/mol), lower row. Green: TFPE-head signal, red: Nuclei staining with DRAQ5. Scale bar, 20 µm, applies to all. Experiments were done in PBS buffer. (**B**) Fusion efficiencies. Whiskers indicate standard deviations of at least three independent experiments. In total, more than 1500 cells were analyzed at each condition.

Upon variation of temperature, two main trends were observed. Liposomes containing DOPE as neutral lipid homogenously stained the plasma membranes of CHO cells in the whole temperature range from 4 °C to 37 °C (see Figure 2A). Here, fusion efficiencies of above 80% were determined from fluorescent micrographs (Figure 2B). In contrast, liposomes containing a phosphocholine, here DOPC, as neutral lipid stuck to the cell surface resulting in an inhomogeneous dotted fluorescence pattern, and afterward were taken up by endocytosis. Internalization by endocytosis was detected in the whole temperature range from 4 °C to 37 °C (Figure 2B). Despite the temperature shock inherent in the procedure, no indications for cell stress were observed.

In all cases, the headgroup of the neutral lipid, PE or PC, respectively, controlled fusion ability, while chain length or saturation had much lower effects (see Table 2). Moreover, the replacement of the head labeled lipid (TFPE-head) as the aromatic component with a chain labeled lipid (TFPE-chain) or a fluorescent lipid analog (DiR) did not significantly influence fusion efficiency of liposomes in the analyzed temperature range (see Table S1).

### 3.2. Phase States of Endocytic and Fusogenic Liposomes

**Figure 3:**
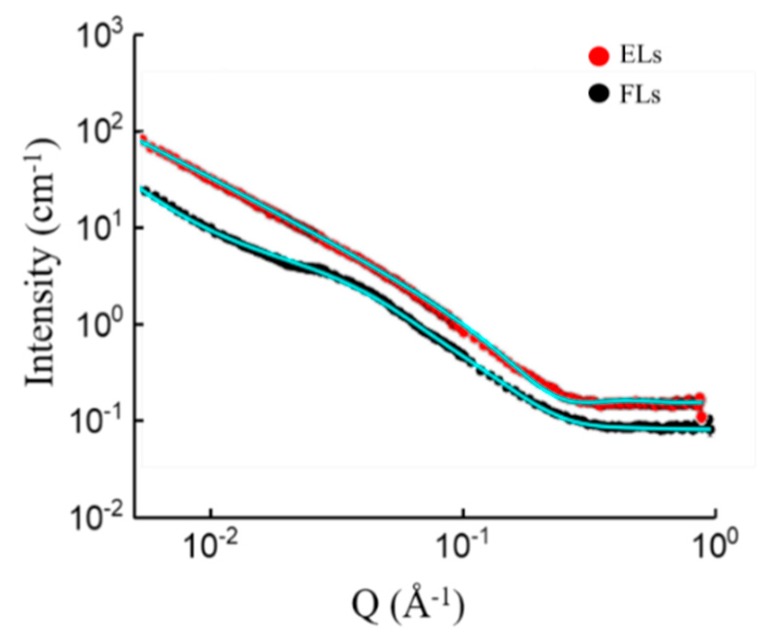
Scattering curves of DOPE/DOTAP/BODIPY-FL-DHPE (1/1/0.1 mol/mol) (**black circles**) liposomes and DOPC/DOTAP/BODIPY-FL-DHPE (1/1/0.1 mol/mol) (**red circles**) liposomes measured at 37 °C. Cyan lines indicate corresponding fits of a single measurement.

To find the reasons underlying the very different fusion behaviors of PC and PE containing cationic liposomes, SANS analyses were carried out at the physiologically most relevant temperature of 37 °C. For these measurements, liposomes were formed in HEPES buffered heavy water (cf. Materials and methods for details). With this method, multi-dispersed, multilayered vesicles were formed. Experimental results and fits are shown in Figure 3. 

The scattering profile of DOPC containing liposomes was adequately modeled by a lamellar lipid phase, with a bilayer thickness of 42.7 Å. DOPE containing liposomes, however, displayed a different scattering pattern with a characteristic shoulder at Q = 0.015 Å^−1^. The latter indicated the presence of small scale features and could not be described by a lamellar phase alone. The best fit was achieved by a superposition of the scattering function of ellipsoid particles and a power law. The curve of DOPE containing liposomes could not be fitted with a cylinder model. This argues against the familiar hexagonal/inverted hexagonal phase of DOPE. Also, the combination of the cylinder model combined with ellipsoid failed to fit the curve. Additionally, combinations of models (lamellar with ellipsoid and lamellar with power law) did not fit perfectly the specific region at Q = 0.015 Å^−1^. Apart from that part of the scattering curve, the lamellar model fitted nicely, which indicates the presence of bilayers. The observed power law contribution is a common occurrence if the probed length scale is smaller than the scattering object; it reflects local structures of the object [33]. Therefore, the agreeable fit of this model combining a power law with an ellipsoid and separate fitting of most of the pattern by the lamellar model leads to the following hypothesis on the structure: We propose small micelle-like structures are embedded into the lipid bilayers. The best fitting power-law exponent was 2.99, which can be attributed to large solid vesicles with a rough surface. The best fitting ellipsoidal particles had a polar radius of 24.8 Å and an equatorial radius of 88.5 Å. The fitting functions are described in the section Materials and Methods.

### 3.3. Influence of Ionic Concentration

The influence of the surrounding ionic concentration was examined for various ionic strengths of the medium. For the purpose of these experiments, the lipid film was hydrated in ultrapure water instead of a buffer to avoid the presence of ions in the liposomal stock solution. Subsequently, liposomes were diluted in phosphate buffer (PB) at low total ion concentration (30 mM) or in phosphate buffer containing additional saline (PBS) at high total ion concentration (280 mM). The presence of ions drastically increased the hydrodynamic size of both types of liposomes (compare Table 1 and Table 3) and reduced the liposomal homogeneity, as shown in Table 3. No significant differences were detected between FLs and ELs (Table 1). The analysis of liposomal zeta potential showed a significant reduction in liposomal charges of both liposomes in the presence of PBS and a moderate decrease in PB buffer compared to glucose solutions.

**Figure 4:**
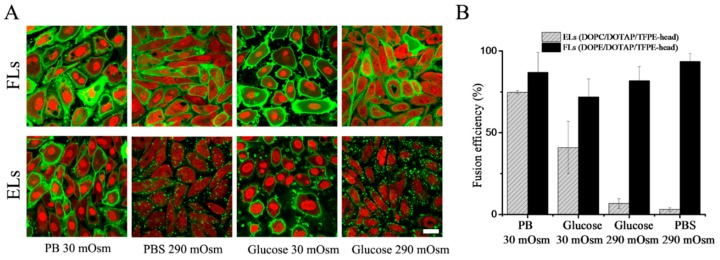
(**A**) Fluorescence micrographs of CHO cells after treatment with fusogenic (DOPE/DOTAP/TFPE-head 1/1/0.1 mol/mol) upper row, and endocytic liposomes, (DOPC/DOTAP/TFPE-head 1/1/0.1 mol/mol) lower row. Green: TFPE-head signal, red: Nucleic staining with DRAQ5. Scale bar, 20 µm, applies to all. (**B**) Fusion efficiencies. Whiskers indicate standard deviations of at least three independent experiments.

Fusion efficiency of CHO cells with the same liposomes was also determined. The fluorescence signal of liposomes after internalization was monitored by confocal microscopy. Liposomes containing DOPE as neutral lipid diluted in PB buffer showed homogeneous membrane staining with high fusion efficiencies of approximately 90%. When the same liposomes were diluted in PBS buffer, they remained fusogenic with similar or slightly higher efficiencies (Figure 4). However, when DOPE was replaced with DOPC as a neutral component, liposomes displayed different behaviors depending on ionic strength. In PB, they fused with CHO cells with high efficiencies, while no significant fusion was detected in PBS (Figure 4). During the treatment with low ionic strength, cells reacted on these hypo-osmotic conditions by membrane blebbing. Nevertheless, cells recovered immediately after the treatment without any signs of damage. The same trends were observed in the case of all investigated PE or PC containing liposomes irrespective of chain length or unsaturation of the neutral component (see Table 4) or exchange of the aromatic component (see Table S2).

### 3.4. Influence of Osmolality

To test if the remarkably different behavior of DOPC containing liposomes in PB and PBS originated from electrostatic or osmotic effects, we varied osmolality by an uncharged solute. To this end, liposomes were prepared as previously described and diluted subsequently in a low (30 mOsm/kg) or a high (290 mOsm/kg) osmolality glucose solution without any addition of salts. Liposomes containing DOPE as neutral lipid diluted in 30 mOsm/kg or 290 mOsm/kg glucose solutions fused with the cell membrane of CHO cells with similar high efficiencies of approximately 80%. In contrast, liposomes containing DOPC as neutral component again showed fusion efficiencies that depended on osmolality. If such liposomes were diluted in 30 mOsm/kg glucose solution, significant fusion (approx. 50% efficiency) was detected, while almost no fusion events were observed in 290 mOsm glucose solution (see Figure 4 and Table 2). During the treatment with low osmolality buffer, cells reacted on hypo-osmotic conditions by membrane blebbing. Nevertheless, after the treatment, cells recovered immediately without any obvious damage. Upon exchange of neutral lipids with various chain lengths and saturation, our results indicated a universal trend valid for liposomes containing PE or PC neutral lipids, as shown in Table 2. PE-containing liposomes fused very efficiently with the plasma membrane of CHO cells independent of chain length, saturation, or the aromatic component (see also Table S2), while the fusion efficiency of PC containing liposomes strongly depended on buffer osmolality. 

### 3.5. Influence of pH 

Both types of liposomes, fusogenic and endocytic, were characterized in the pH range of buffer between 5 and 9. Even though, the PBS buffer capacity was not ideal in the whole range, all experiments were carried out in a PBS buffer, where the pH was adjusted to the appropriate value. FLs (DOPE/DOTAP/TFPE-head 1/1/0.1 mol/mol), as well as ELs (DOPC/DOTAP/TFPE-head 1/1/0.1 mol/mol), became less homogeneous with larger particles formed at higher pH values. Zeta potential of FLs was reduced when pH was increased, while ELs did not show any pH-dependent changes (see Table 5).

**Figure 5:**
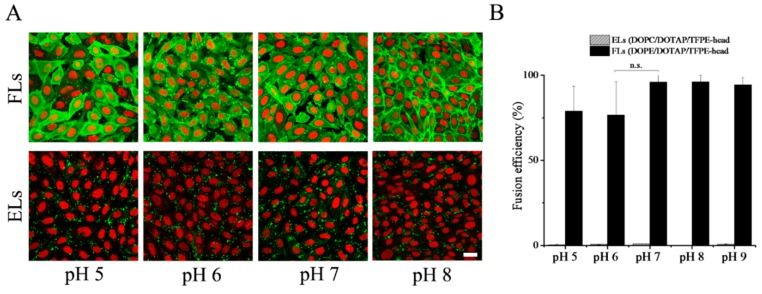
(**A**) Fluorescence micrographs of CHO cells after treatment with fusogenic (DOPE/DOTAP/TFPE-head, 1/1/0.1 mol/mol), upper row, and endocytic liposomes (DOPC/DOTAP/TFPE-head, 1/1/0.1 mol/mol), lower row, at different pH values. Green: TFPE-head signal, red: Nucleic staining with DRAQ5. Scale bar, 20 µm, applies to all. (**B**) Fusion efficiencies. Whiskers indicate standard deviations of at least three independent experiments.

We also analyzed the pH dependence of membrane fusion of cationic liposomes with CHO cells at 37 °C. Liposomes containing PE, here DOPE, as neutral lipid homogenously stained the cellular plasma membrane of CHO cells at all pH values in the range from 5 to 9. Throughout the whole range, tested fusion efficiency exceeded 75% (Figure 5). In contrast, liposomes containing phosphocholine, here DOPC, as neutral lipid adhered to the cell surface, which resulted in an inhomogeneous speckled fluorescence pattern. In the whole pH range from 5 to 9, internalization by endocytosis was rarely detected with a fusion efficiency above 1% (Figure 5).

## 4. Discussion

In the absence of fusogenic peptides or proteins, membrane fusion occurs in the following sequence [4,13,18,34,35]. First, the two membranes must be brought into close contact. This step requires the removal of tightly bound hydration water. Second, the locally disrupted outer membrane leaflets melt together, forming hemifusion intermediate structures called membrane stalks. Third, the inner lipid monolayers reorganize, which results in pore opening, as well as full membrane and content mixing. Each of the intermediate states is characterized by a well-defined free enthalpy that together defines the activation energy barrier for fusion. Some chemical compounds, such as ions, drugs, or distinct lipids, as well as conditions like temperature, pH, or osmolality of the buffer, can alter this barrier [18,35]. Because we recently analyzed the influence of the chemical composition of cationic liposomes on their fusion efficiency with mammalian cell membranes [25], we tackled here the effect of environmental conditions on fusion efficiency.

Changes of the environmental conditions such as ionic concentration, pH, or buffer osmolality mainly influence the first step in the fusion process. For overcoming the first barrier and bringing the two membranes into close contact, the reduction of the water interface between the fusion partners is mandatory. For example, ions bound to the membranes (Ca^2+^, Na^+^, and K^+^) can modify their surface polarities, which in turn reduces the hydration-dependent intermembrane repulsion [34,36,37,38]. Nevertheless, our results showed that in the case of FLs, fusion efficiency was not influenced by the ionic composition of the surrounding buffer, suggesting the presence of another factor that is more important for the fusion process than the electrostatic interaction. Additionally, even though we observed no differences between size or zeta potential of fusogenic and endocytic liposomes, both types of liposomes were taken up by very different cellular pathways. Also, model membrane experiments by other groups demonstrated that a mixture of giant unilamellar vesicles (GUVs) with opposite surface charge (e.g., DOTAP containing liposomes and DOPS containing liposomes) aggregate readily and the lipid mixing efficiency does not change with increasing ionic strength [39,40]. In the case of ELs, however, the ionic environment played a crucial role in membrane fusion induction. Here, an increased fusion efficiency was detected only at a low salt concentration (30 mOsm/kg). At physiological salt concentration (280 mOsm/kg), the fusion efficiency was reduced again.

Additionally, the same trend was observed when buffer osmolality was changed from low to high (from 30 mOsm/kg to 280 mOsm/kg) by adding sugar instead of salt. The only exception of PC containing liposomes showing elevated fusion capacity were liposomes with DSPC. This abnormal fusion behavior can most likely be explained by the particular phase state of DSPC in the presence of phospholipids with notably different properties. For example, DSPC mixed with DMPC forms a non-ideal mixture with a broad gel-fluid coexisting region [41,42]. We assume that such a phase coexistence, also present in a DOTAP/DSPC mixture, is favorable for the formation of fusion intermediates. Despite their extraordinary high-fusion ability, DSPC containing liposomes still showed the same trend of higher fusion efficiency at low osmolality and ionic strength buffers compared to physiological conditions. Therefore, we suggest that the increased membrane fusion was caused by osmotic destabilization of CHO cells, rather than by the ionic interaction between the liposomal and the cellular membrane. In this context, the following recent observation is of interest. Middel et al. showed that repair of membrane lesions in skeletal muscle is accompanied by transiently increased concentrations of negatively charged phosphatidylserine lipids [43]. A similar mechanism might cause enhanced electrostatic attraction in osmotically stressed cell membranes.

After a close contact of the two membranes, a transient disturbance of the bilayers structure and subsequent reorganization is required to overcome the energy barrier of the different steps and form hemifusion intermediates [44]. It has been proposed that the phase transition between a lamellar (L) and an inverted hexagonal (H_II_) phase is essential for the formation of such intermediate structures [45,46,47]. Several molecules have been described as initiators for the H_II_ phase, such as drugs, surfactants, and lipids, e.g., PEs. However, for our fusogenic lipid mixture that contains DOPE, SANS measurements are not compatible with a H_II_ phase (Figure 2), but rather suggest a mixture of lamellar membranes with membrane compartments of high curvature (with polar radii of 24.8 Å and an equatorial radius of 88.5 Å, see Figure 2). Bulavin and Lebovka reported similar fitting models for rough interfaces of microcapsules carrying five or eight polymer bilayers [33]. We propose here that membrane fusion is facilitated by the observed micelle-like inclusions in the membranes. As they exhibit curvatures of similar magnitude than lipid H_II_ phases, we expect an effect of comparable size. Because the fusogenic lipid mixture studied here can fuse with any biological membrane [21] without any influence of physical conditions such as temperature, osmolality, ionic strength, or pH of the buffer, as shown above, we hypothesize that the observed structure generally occurs for this lipid mixture and causes the observed high fusogenicity. Lipid mixtures in the lamellar phase, here the ELs, have been found to be non-fusogenic.

For a better understanding of the second and the third steps of membrane fusion, we have to take into consideration the effective shape of the membrane-forming molecules [13]. Lipids with an effective cylindrical shape (e.g., PC) form bilayers with zero spontaneous curvature. Lipids obtaining inverted conical shapes lead to positive membrane curvature, while lipids with conical effective molecular shape (e.g., PE) form membrane structures with negative curvature. Such membranes are postulated as more fusogenic [48,49]. Our results corroborate this hypothesis, namely, that the lipid composition has a high impact on liposomal behavior [25]. Here, we found that cationic liposomes containing PEs with a conical effective molecular shape fuse with the highest efficiency with the plasma membrane of CHO cells, therefore, they are assigned as fusogenic, while PC containing cationic liposomes remain rather non-fusogenic. Similar behavior was also found for PE and PC containing cationic liposomes when they were incubated with red blood cells [50]. The fact that such FLs do not show any considerable differences in fusion efficiency with cells depending on the temperature, osmolality, or ionic concentration of the buffer indicates that the presence of a 3D lipid phase formed by spherical membrane structures with high curvatures lowers the energy barrier for fusion significantly, and thus, efficiently facilitates membrane fusion. In this context, the additional structures within the vesicle lamellae can be considered a pre-formation of the intermediate structures that are necessary for the lamellar fusion. Since they are already present, they do not need to be formed during the fusion process, and therefore, lower the barrier for the occurrence of fusion.

## 5. Conclusions

For a lipid formulation that fuses very efficiently with cell membranes, we find a structure characterized by the simultaneous presence of lipid bilayers and small micelle-like structures with high surface curvatures. We propose that this peculiar structure is present at a broad range of conditions and gives rise to efficient fusion. In contrast, for lipids that are mainly organized in a lamellar phase, like the endocytic liposomes analyzed in our study, buffer conditions strongly influence membrane fusion. However, under physiological conditions, overall fusion efficiencies remain very low.

## Figures and Tables

**Table 1:** Temperature dependence of the hydrodynamic diameter, polydispersity index (PDI), and zeta potential of fusogenic (DOPE/DOTAP/TFPE-head 1/1/0.1 mol/mol) (FLs) and endocytic (DOPC/DOTAP/TFPE-head 1/1/0.1 mol/mol) (ELs) liposomes. Experiments are performed in a PBS buffer at physiological pH 7.4. Given are averages over three independent measurements and, in parentheses, their standard deviations.

Liposomal Type	Hydrodynamic Diameter (nm) (s.d.)			
	4 °C	20 °C	30 °C	37 °C
(FLs)	110 (13)	116 (13)	118 (16)	117 (13)
(ELs)	142 (42)	154 (68)	158 (71)	155 (72)
	PDI (s.d.)			
(FLs)	0.26 (0.06)	0.23 (0.02)	0.23 (0.02)	0.23 (0.02)
(ELs)	0.34 (0.12)	0.32 (0.11)	0.35 (0.11)	0.33 (0.11)
	Zeta Potential (mV) (s.d.)			
(FLs)	59 (4)	48 (8)	51 (3)	43 (13)
(ELs)	68 (13)	64 (10)	56 (8)	58 (8)

**Table 2:** Temperature dependence of fusion efficiencies of liposomes containing the cationic lipid DOTAP, different neutral lipids, and TFPE-head as an aromatic molecule (1/1/0.1 mol/mol). Experiments are performed in a PBS buffer at physiological pH 7.4 and osmolality 280 mOsm/kg. Given are averages over at least three independent measurements and, in parentheses, their standard deviations.

Liposomal Composition	Fusion Efficiency % (s.d.)			
	4 °C	20 °C	30 °C	37 °C
C16(0)PE/DOTAP/TFPE-head	97 (1)	98 (2)	98 (1)	99 (1)
C16(0)PC/DOTAP/TFPE-head	0 (0)	0 (0)	0 (0)	0 (0)
C16(1)PE/DOTAP/TFPE-head	88 (11)	93 (6)	96 (3)	98 (2)
C16(1)PC/DOTAP/TFPE-head	13 (3)	16 (7)	14 (3)	17 (1)
C18(0)PE/DOTAP/TFPE-head	92 (6)	96 (1)	89 (9)	95 (5)
C18(0)PC/DOTAP/TFPE-head	37 (8)	38 (11)	47 (6)	62 (5)
C18(1)PE/DOTAP/TFPE-head	83 (9)	90 (3)	87 (4)	92 (6)
C18(1)PC/DOTAP/TFPE-head	3 (2)	5 (4)	2 (1)	4 (2)

**Table 3:** Ionic concentration dependence of the hydrodynamic diameter, polydispersity index (PDI) and zeta potential of fusogenic (DOPE/DOTAP/TFPE-head 1/1/0.1 mol/mol) (FLs) and endocytic (DOPC/DOTAP/TFPE-head 1/1/0.1 mol/mol) (ELs) liposomes. Experiments are performed in a PBS buffer at physiological pH 7.4. Given are averages over three independent measurements and, in parentheses, their standard deviations.

Liposomal Type	Hydrodynamic Diameter (nm) (s.d.)			
	PB (30 mOsm/kg)	PBS (290 mOsm/kg)	Glucose (30 mOsm/kg)	Glucose (290 mOsm/kg)
(FLs)	568 (145)	567 (102)	537 (71)	493 (157)
(ELs)	460 (277)	551 (357)	524 (296)	515 (357)
	PDI (s.d.)			
(FLs)	0.25 (0.05)	0.36 (0.13)	0.41 (0.09)	0.32 (0.01)
(ELs)	0.34 (0.07)	0.32 (0.11)	0.35 (0.11)	0.33 (0.11)
	Zeta Potential (mV) (s.d.)			
(FLs)	36 (6)	28 (4)	72 (3)	69 (11)
(ELs)	62 (1)	36 (1)	78 (3)	73 (7)

**Table 4:** Fusion efficiencies of liposomes containing the cationic lipid DOTAP, different helper lipids, and TFPE-head as a dye molecule (1/1/0.1 mol/mol) depending on the osmolality of the solution at 37 °C. Average values of at least three independent experiments and their standard deviations are given.

Liposomal Composition	Fusion Efficiency % (s.d.)			
	PB (30 mOsm/kg)	PBS (290 mOsm/kg)	Glucose (30 mOsm/kg)	Glucose (290 mOsm/kg)
**C16(0)PE/DOTAP/TFPE-head**	98 (1)	85 (5)	95 (4)	79 (2)
**C16(0)PC/DOTAP/TFPE-head**	93 (6)	3 (3)	66 (2)	5 (4)
**C16(1)PE/DOTAP/TFPE-head**	86 (4)	99 (1)	98 (1)	99 (1)
**C16(1)PC/DOTAP/TFPE-head**	33 (3)	0 (0)	27 (7)	10 (2)
**C18(0)PE/DOTAP/TFPE-head**	76 (9)	89 (2)	82 (12)	98 (2)
**C18(0)PC/DOTAP/TFPE-head**	89 (3)	51 (1)	64 (8)	65 (8)
**C18(1)PE/DOTAP/TFPE-head**	87 (12)	94 (5)	72 (11)	82 (8)
**C18(1)PC/DOTAP/TFPE-head**	75 (1)	3 (1)	41 (16)	7 (3)

**Table 5:** pH dependence of the hydrodynamic diameter, polydispersity index (PDI) and zeta potential of fusogenic (DOPE/DOTAP/TFPE-head 1/1/0.1 mol/mol) (FLs) and endocytic (DOPC/DOTAP/TFPE-head 1/1/0.1 mol/mol) (ELs) liposomes. Experiments were performed in a PBS buffer titrated to the indicated pH. Given are averages over three independent measurements and, in parentheses, their standard deviations.

Liposomal Type	Hydrodynamic Dyameter (nm) (s.d.)				
	pH 5	pH 6	pH 7	pH 8	pH 9
(FLs)	221 (193)	179 (124)	177 (36)	276 (52)	333 (117)
(ELs)	118 (24)	117 (19)	108 (23)	186 (22)	197 (16)
		PDI (s.d.)			
(FLs)	0.30 (0.10)	0.30 (0.11)	0.35 (0.11)	0.31 (0.21)	0.35 (0.21)
(ELs)	0.21 (0.03)	0.22 (0.02)	0.19 (0.02)	0.23 (0.09)	0.32 (0.18)
		Zeta Potential (mV) (s.d.)			
(FLs)	42 (7)	39 (2)	36 (4)	26 (4)	15 (5)
(ELs)	35 (4)	36 (3)	38 (3)	35 (3)	38 (5)

## Supplementary Materials

The following are available online at https://www.mdpi.com/2079-4991/9/7/1025/s1, Figure S1: Fluorescence and phase contrast micrographs of CHO cells upon treatment with fusogenic (FLs) (DOPE/DOTAP/TFPE-head 1/1/0.1 mol/mol) and endocytic (ELs) (DOPC/DOTAP/TFPE-head 1/1/0.1 mol/mol) liposomes, Table S1: Temperature dependence of the fusion efficiencies of liposomes containing the cationic lipid DOTAP, different helper lipids, and TFPE-chain or DiR as an aromatic molecule (1/1/0.1 mol/mol), Table S2: Fusion efficiencies of liposomes containing the cationic lipid DOTAP, different helper lipids, and TFPE-chain or DiR as dye molecule (1/1/0.1 mol/mol) depending on the osmolarity and ionic strength of the buffer.
