# Supplementary material for: Influence of Environmental Conditions on the Fusion of Cationic Liposomes with Living Mammalian Cells
Source: Nanomaterials (Basel). 2019 Jul 17;9(7):1025. doi: 10.3390/nano9071025 (PMC6669649; doi:10.3390/nano9071025)
Supplement: Supplementary file 1 [file nanomaterials-09-01025-s001.pdf]

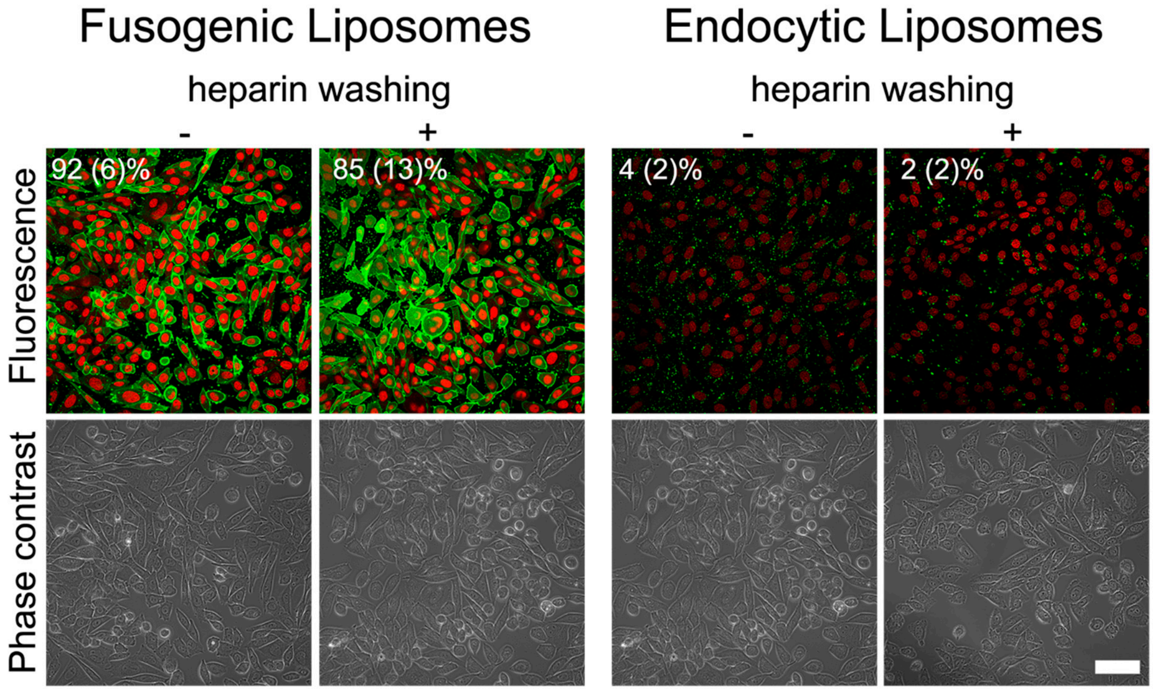

Figure S1. Fluorescence and phase contrast micrographs of CHO cells upon treatment with fusogenic (FLs) (DOPE/DOTAP/TFPE-head 1/1/0.1 mol/mol) and endocytic (ELs) (DOPC/DOTAP/TFPE-head 1/1/0.1 mol/mol) liposomes. Cellular intercalation of the liposomes was verified by an additional washing step with a heparine solution. Uptake efficiencies are shown in the micrographs (%). Green: TFPE-head signal, red: nucleic staining with DRAQ5. Scale bar, 50 µm.

Table S1. Temperature dependence of the fusion efficiencies of liposomes containing the cationic lipid DOTAP, different helper lipids, and TFPE-chain or DiR as an aromatic molecule (1/1/0.1 mol/mol). Efficiencies of endocytosis and fusion give a sum of 100%. Average values of at least three independent measurements and their standard deviations are given.

**Liposomal composition**	**Fusion efficiency % (s.d.)**			
	**4°C**	**20°C**	**30°C**	**37°C**
**C16(0)PE**/DOTAP/TFPE-chain	84 (14)	93 (7)	99 (0)	97 (2)
**C16(0)PC**/DOTAP/TFPE-chain	4 (1)	0 (0)	0 (0)	0 (0)
**C16(1)PE**/DOTAP/TFPE-chain	84 (14)	94 (6)	99 (1)	97 (2)
**C16(1)PC**/DOTAP/TFPE-chain	15 (3)	14 (7)	13 (6)	15 (6)
**C18(0)PE**/DOTAP/TFPE-chain	97 (3)	89 (11)	98 (3)	93 (5)
**C18(0)PC**/DOTAP/TFPE-chain	53 (25)	59 (13)	69 (3)	55 (14)
**C18(1)PE**/DOTAP/TFPE-chain	81 (3)	83 (10)	94 (5)	90 (7)
**C18(1)PC**/DOTAP/TFPE-chain	3 (4)	0 (0)	3 (3)	0 (0)
**C16(0)PE**/DOTAP/DiR	86 (11)	94 (2)	92 (2)	97 (1)
**C16(0)PC**/DOTAP/DiR	7 (4)	3 (1)	9 (3)	5 (4)
**C16(1)PE**/DOTAP/DiR	95 (8)	86 (7)	97 (3)	97 (4)
**C16(1)PC**/DOTAP/DiR	4 (2)	5 (3)	1 (1)	1 (1)
**C18(0)PE**/DOTAP/DiR	95 (5)	98 (1)	85 (1)	94 (5)
**C18(0)PC**/DOTAP/DiR	16 (6)	17 (3)	12 (3)	13 (5)
**C18(1)PE**/DOTAP/DiR	80 (5)	98 (1)	99 (1)	94 (9)
**C18(1)PC**/DOTAP/DiR	4 (1)	1 (1)	8 (1)	2 (1)

Table S2. Fusion efficiencies of liposomes containing the cationic lipid DOTAP, different helper lipids, and TFPE-chain or DiR as dye molecule (1/1/0.1 mol/mol) depending on the osmolarity and ionic strength of the buffer. Efficiencies of endocytosis and fusion give a sum of 100%. Average values of at least three independent measurements and their standard deviations are given.

**Liposomal composition**	**Fusion efficiency % (s.d.)**			
	**PB 30 mOsm**	**PBS 290 mOsm**	**Glc 30 mOsm**	**Glc 290 mOsm**
**C16(0)PE**/DOTAP/TFPE-chain	84 (13)	89 (10)	96 (2)	99 (1)
**C16(0)PC**/DOTAP/TFPE-chain	83 (5)	1 (0)	97 (3)	3 (2)
**C16(1)PE**/DOTAP/TFPE-chain	60 (16)	95 (8)	83 (2)	99 (1)
**C16(1)PC**/DOTAP/TFPE-chain	84 (8)	5 (2)	44 (22)	5 (1)
**C18(0)PE**/DOTAP/TFPE-chain	49 (9)	75 (13)	86 (9)	65 (10)
**C18(0)PC**/DOTAP/TFPE-chain	70 (16)	52 (6)	62 (5)	56 (6)
**C18(1)PE**/DOTAP/TFPE-chain	87 (12)	89 (5)	89 (6)	99 (1)
**C18(1)PC**/DOTAP/TFPE-chain	59 (3)	3 (1)	56 (3)	7 (3)
**C16(0)PE**/DOTAP/DiR	87 (10)	91 (9)	65 (12)	91 (7)
**C16(0)PC**/DOTAP/DiR	39 (10)	7 (2)	30 (8)	16 (2)
**C16(1)PE**/DOTAP/DiR	71 (29)	97 (3)	76 (20)	97 (2)
**C16(1)PC**/DOTAP/DiR	66 (13)	8 (6)	31 (13)	3 (2)
**C18(0)PE**/DOTAP/DiR	70 (5)	89 (5)	97 (1)	98 (1)
**C18(0)PC**/DOTAP/DiR	68 (12)	30 (8)	66 (8)	68 (11)
**C18(1)PE**/DOTAP/DiR	90 (5)	97 (3)	87 (1)	70 (9)
**C18(1)PC**/DOTAP/DiR	83 (2)	2 (1)	49 (5)	0 (0)
